# Evaluation of the Effects of Thermal Aging on the Surface Roughness of Novel Tooth-Colored Restorative Materials

**DOI:** 10.3390/dj12120390

**Published:** 2024-12-03

**Authors:** Austin Galbraith, Neamat Hassan Abubakr

**Affiliations:** 1 DMD Student School of Dental Medicine, University of Nevada, Las Vegas, NV 89557, USA; galbra4@unlv.nevada.edu; 2 Department of Biomedical Sciences, School of Dental Medicine, University of Nevada, Las Vegas, NV 89557, USA

**Keywords:** ormocer, resin-modified glass ionomer, glass ionomer hybrid, resin composite, tooth-colored materials

## Abstract

**Background:** The development of composite resins has led to novel materials aimed at improving restoration longevity. This study evaluates the surface roughness of four tooth-colored restorative materials after thermal aging. **Methods:** Eighty Class V preparations were restored with Admira Fusion, Beautifil II, Equia Forte HT, and Filtek. The samples underwent thermocycling, and their surface roughness was measured with a 3D non-contact profilometer at 24 h post-restoration and after simulation for 1, 3, and 5 clinical years. **Results:** Equia Forte HT showed the highest surface roughness and significant surface deterioration over time, while Admira Fusion maintained the lowest roughness across all intervals. **Conclusions:** Admira Fusion, Filtek, and Beautifil II demonstrated superior surface stability, with Equia Forte HT showing the least favorable performance.

## 1. Introduction

The development of tooth-colored restorative materials has rapidly gained popularity due to their aesthetics and bonding ability [1]. Some dental materials exhibit superior properties relative to others, particularly in terms of surface roughness, which can significantly impact the long-term survival of restorations. Although many tooth-colored restorative materials are based on a single type of resin, hybrid materials (resin and glass ionomers) integrate advantageous characteristics from multiple resins, resulting in enhanced overall performance [2]. Despite numerous claims regarding their superior qualities, these materials must be evaluated under various conditions to assess their true durability.

Several new hybrid restorative materials are available for clinical use; examples include nano-hybrid ormocers that consist of a ceramic base, Surface Pre-reacted Glass Ionomers (S-PRGs), and hybrid glass ionomers [3,4,5].

“Ormocer” is an abbreviation for “organically modified ceramic”. It features a methacrylate-free matrix formulation developed for applications in science and technology. Ormocers are distinct from conventional composites due to their matrix, which incorporates both organic and inorganic components [6,7]. The synthesis of the ormocer matrix is a three-dimensional cross-linked copolymer structure [8]. The introduction of ormocers in dentistry was achieved by combining ormocer technologies with nanohybrid materials, which, as a result, significantly enhanced the bonding compatibility of the material when compared to traditional composites [9]. This is attributed to the high degree of cross-linking between the chemical elements within the matrix, providing superior mechanical properties and durability [9,10]. A prominent example of a dental ormocer is Admira Fusion (VOCO GmbH), introduced in 2015. This material is notable for its pure ormocer matrix chemistry, which excludes conventional dimethacrylates [6,11]. The ceramic base of Admira Fusion (ormocer) offers high biocompatibility and features a universal shade with a chameleon effect, enabling it to blend seamlessly with adjacent tooth structures [3].

The term “giomer” refers to a class of restorative materials in dentistry that integrates the beneficial properties of glass ionomer cement, such as fluoride release, with the superior aesthetics and long-term clinical performance of composite resins. [12,13]. Giomer restorative materials have been one of the most reliable restorative options over the last years [13]. The production of giomer materials involves acid-reactive, fluoride-containing glass particles that react with poly acids in the presence of water. This reaction forms a durable matrix that releases fluoride and enhances the material’s structural integrity and longevity [12,13]. The formulation of giomers is based on Pre-Reacted Glass Ionomer (PRG) technology, which involves pre-reacting fluoro-aluminosilicate glass fillers with polyacrylic acid. This reaction forms a stable glass ionomer phase called a wet siliceous hydrogel. The resulting glass ionomer is then freeze-dried, milled, silane-treated, and ground to form the PRG fillers [14]. Giomer restorative materials provide several unique advantages, including enhanced wear resistance, high radiopacity, and continuous fluoride release and recharge capabilities. These benefits are attributed to the stable glass ionomer phase formed before integrating into the resin matrix [15]. Importantly, unlike other fluoride-containing dental adhesives, the fluoride ion release in giomers does not lead to material degradation. This unique feature, where the release mechanism mirrors that of conventional glass ionomers, makes giomers a superior and intriguing choice for dental restorations, reassuring dental professionals [14]. PRG fillers are categorized into surface-reaction PRG (S-PRG) fillers and full-reaction PRG (F-PRG) fillers. In F-PRG fillers, the entire filler particle reacts with polyacrylic acid, resulting in a higher fluoride release as the particle’s core is completely reacted. This high fluoride release can be beneficial in certain cases, but it also means that F-PRG fillers degrade more rapidly than S-PRG fillers [4,12,15]. This rapid degradation could affect the restoration’s longevity, as the filler may lose its structural integrity sooner. In contrast, S-PRG fillers undergo surface reactions with polyacrylic acid, leaving the glass core intact, slowing the degradation process, and potentially leading to longer-lasting restorations [14]. Beautifil II incorporates S-PRG technology, which facilitates fluoride recharge and provides anti-plaque effects by integrating the benefits of both glass ionomer and conventional composite materials [4,13].

A recently introduced restorative material that gained clinical acceptability due to its enhanced mechanical and biomimetic properties was the high-viscosity glass ionomer cement (HVGIC) [16]. The development of HVGIC addresses the mechanical limitations of traditional glass ionomer cement (GIC) by offering improved physical characteristics such as flexural, compressive, and tensile strengths, along with superior wear resistance [17,18]. One notable example of HVGIC is the EQUIA^®^ system, introduced by GC Corporation in 2009, followed by the next-generation EQUIA Forte^®^ in 2014 [19]. Recent clinical trials have demonstrated that HVGIC materials, such as Equia Forte Fil (GC America), yield excellent outcomes in both permanent and primary teeth [20,21,22,23]. A recently introduced and enhanced high-viscosity glass ionomer (HVGI) material, Equia Forte HT, offers significant advancements over its predecessor. This system incorporates a higher molecular weight polyacrylic acid, contributing to improved structural integrity, and it features a novel coating agent containing an advanced monomer that provides enhanced protection during the critical early maturation phase [24]. Additionally, the particle size distribution of Equia Forte HT has been optimized compared to the earlier Equia Forte formulation [24]. These improvements have increased flexural and compressive strength, attributed to superior matrix loading, thereby expanding its suitability for stress-bearing and non-stress-bearing restorations [25]. This comparison provides dental professionals with confidence in the effectiveness of HVGIC [20,25,26]. Equia Forte HT glass ionomer has properties including no polymerization shrinkage, excellent marginal seal, and fluoride release for up to 6 months [24]. Many of these products have new features that will be beneficial, but the surface profile remains of utmost importance to the life and overall performance of the restoration.

One recent advancement in composite resin restorations is the Filtek™ Supreme Ultra Universal Restorative, a dental composite material designed for anterior and posterior restorations. It offers a combination of esthetics, strength, and polish retention. Available in 36 shades and four opacities—dentin, body, enamel, and translucent—it ensures precise color matching to natural dentition.

In the oral cavity, rough surfaces facilitate plaque formation and maturation, which leads to greater bacterial accumulation and often contributes to a progression towards disease [27]. An optimal surface profile is essential for a restorative material’s capacity to endure the masticatory forces encountered in stress-bearing regions of the oral cavity [28]. It significantly contributes to the mechanical durability of restorative materials and is critical for minimizing dental plaque accumulation, thereby enhancing the restoration’s clinical aesthetics and longevity [29]. Furthermore, achieving appropriate surface topography is fundamental to any restorative material’s overall success and durability [30].

The comparison between these materials regarding surface roughness could give quantitative evidence for dentists to guide their restorative decisions. Given Filtek Supreme’s widespread use and extensive examination in meta-analyses, it was used as the reference standard for comparison in this study. This study’s hypothesis is that there is no difference in surface roughness of Class V restorations when using novel tooth-colored restorative materials compared to standard resin-based restorative materials. The present study aims to evaluate the effects of thermal aging on the surface roughness of different restorative materials, namely Filtek, Admira Fusion, Beautifil II, and Equia Forte.

## 2. Materials and Methods

**Figure 1:**
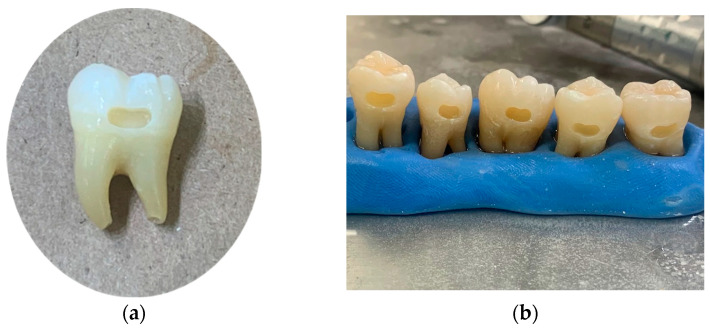
(**a**) Single Class V preparation close-up; (**b**) Class V preparations in putty.

Class V preparations (5 × 2 × 2 mm) were made on the buccal surface of 80 newly extracted molars [Figure 1]. All molars were free of any caries and defects. All cavities were prepared 1 mm above the cementoenamel junction using a high-speed Bien Air handpiece (Bien-Air USA, Inc., Irvine, CA, USA). A 330 bur was used initially, then a 57 straight bur was used to correct undercuts made by the inverse taper of the 330.

The teeth were standardized by measuring each prep with a UNC-15 Perio probe (Hu-FriedyGroup, Chicago, IL, USA) at five different locations: mesiodistal width of 5 mm, cervico-occlusal width of 2 mm at the mesial and distal locations, and a 2 mm depth of the prep at the same mesial and distal locations. A total of 90 teeth were initially prepared, but 10 did not fit the standardization protocol, resulting in a total of 80 preparations. The teeth were randomly divided into four groups, with 20 teeth per group. As discussed, four restorative materials were used: ormocer (Admira Fusion), fluoride-releasing universal nanohybrid composite (Beautifil II), a glass hybrid restorative material (Equia Forte HT), and the standard universal nanoparticle composite (Filtek Sup. Ultra) [Table 1]. Following the manufacturer’s instructions, a bevel in the prepared teeth was included for Beautifil II and Filtek Supreme Ultra. Admira Fusion indicates a bevel only in anterior teeth, which were not used in this study. All restorations were performed following each of the product’s unique manufacturing instructions.

Filtek Restoration Procedure: The preparation for the Filtek restoration was first treated with 3M ESPE Scotchbond Universal Etchant, which was applied for 15 s, followed by a 15 s rinse with water. The tooth was then gently dried, taking care to avoid excessive desiccation of the dentin. Subsequently, 3M ESPE Scotchbond Universal Adhesive was applied to the preparation and scrubbed in for 20 s. The adhesive was lightly air-dried for 5 s to facilitate solvent evaporation before being light-cured for 10 s. The restorative material was then placed, shaped, and light-cured for 20 s.

Equia Forte HT Restoration Procedure: The restoration procedure using Equia Forte HT began with the application of GC Cavity Conditioner, which was left on the cavity surface for 10 s. After rinsing and gently drying the conditioned area, the Equia Forte HT material was mixed by pressing the plunger for 10 s using a dental amalgamator. It was then placed into the capsule applier, primed by clicking the capsule twice, and dispensed within 10 s. The material’s working time is approximately 1 min and 15 s. After the material was added and contoured, Equia Forte HT Coat was applied and light-cured for 20 s.

Admira Fusion Restoration Procedure: The preparation for Admira Fusion restoration was etched with 35% phosphoric acid, then applied to the enamel for 20 s and to dentin for 15 s. The etchant was then rinsed off for 20 s, and the tooth was carefully dried. A thin, even layer of “Admira Bond” (one-component dentine and enamel bond) adhesive was applied, left for 30 s, and gently air-dried with a faint air stream. The adhesive was polymerized using a curing light for 20 s. The Admira Fusion material was placed in the tooth cavity, shaped, and light-cured for an additional 20 s.

Beautifil Restoration Procedure: For the Beautifil restoration, BeautiBond adhesive was applied uniformly to the entire inner surface of the cavity preparation. After a 10 s waiting period, gentle air-drying for 3 s was performed, followed by a more forceful air stream to ensure the adhesive layer was uniformly distributed. The adhesive was light-cured for 5 s. Beautifil material was then dispensed from a syringe using the provided tip, shaped within the cavity preparation, and light-cured for 10 s.

Standardization of Methodology: To minimize potential variability and human error, all cavity preparations, restorations, and data collection were conducted by a single operator. A Kerr Demi Plus curing light (Kerr Corporation, Brea, CA, USA) was used for all light-curing procedures. Finishing was carried out using burs and a Onegloss polishing point, which were used to polish each restorative material post-curing. A putty matrix was employed to prevent cross-contamination between materials and to maintain proper grouping.

**Figure 2:**
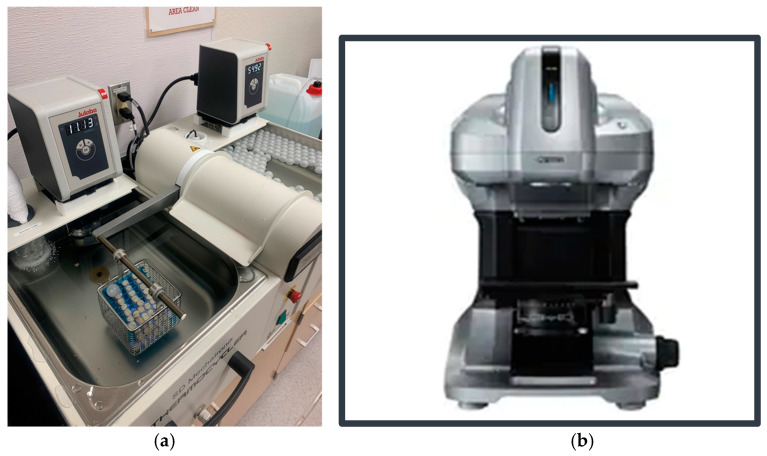
(**a**) Thermal aging machine (SD Mechatronik, Germany); (**b**) 3D Non-Contact Profilometer Wide-area 3D Measurement System (Keyence, Japan).

Surface roughness measurements were conducted using a non-contact 3D profilometer (VR-3100; Keyence, Keyence Corporation, Osaka, Japan), targeting two specific points on each composite restoration and calculating the average surface roughness (Ra) over the total area between those selected points [Figure 2b]. These same points were measured at different intervals following thermal aging. Thermal aging was performed using a thermocycling machine (Thermocycler, SD Mechatronik, Feldkirchen-Westerham, Germany) with pure distilled water baths alternating between 5 °C and 55 °C at 20 s intervals [Figure 2a]. Images and measurements were taken post-restoration, after 10,000, 30,000, and 50,000 cycles, corresponding to 0, 1, 3, and 5 estimated clinical years, respectively [31]. Readings for surface roughness were recorded 24 h post-restoration and after 1, 3, and 5 estimated clinical years. When not in use, the specimens were stored in artificial saliva (Pickering Laboratories, CA, USA) at 37 °C in a temperature-controlled unit, simulating the human body’s internal temperature. Statistical analysis was performed using a one-way analysis of variance (ANOVA) and post hoc Tukey HSD with significance set at *p* < 0.05.

## 3. Results

**Figure 3:**
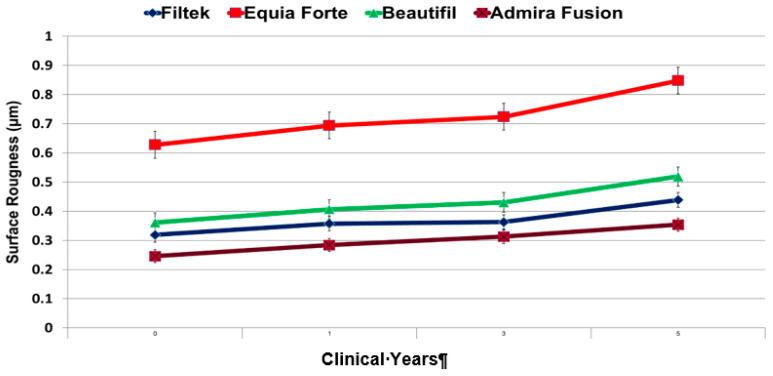
Average surface roughness (Ra) after thermocycling.

All of the examined restorations exhibited a change in the surface profile after five clinical years [Figure 3]. Admira Fusion showed the smallest changes in surface roughness (0.108 µm) when comparing post-restoration to 5 clinical years (or 50,000 thermocycles). Admira Fusion also demonstrated the least overall surface roughness (on average) for each measurement cycle.

Equia Forte HT showed the most significant surface roughness deterioration change (*p*-value = 0.001) after 5 clinical years (0.22 µm), as well as the highest overall surface roughness (on average) for each clinical year [Table 2]. Using a one-way ANOVA with a post hoc Tukey HSD test, the results showed that the difference between Admira Fusion and the Equia Forte HT was highly significant across the different time intervals [Table 2].

Using a one-way ANOVA with post hoc Tukey HSD test, the results indicated a highly significant difference (*p* < 0.001) between group C and the other materials examined across the different time intervals [Table 2]. This change is also visually evident in Figure 3, where the graph illustrates an increase in surface roughness for all materials after five clinical years. Notably, there was a marked increase in surface roughness between the third and fifth years for three materials: C, B, and D [Figure 3].

**Figure 4:**
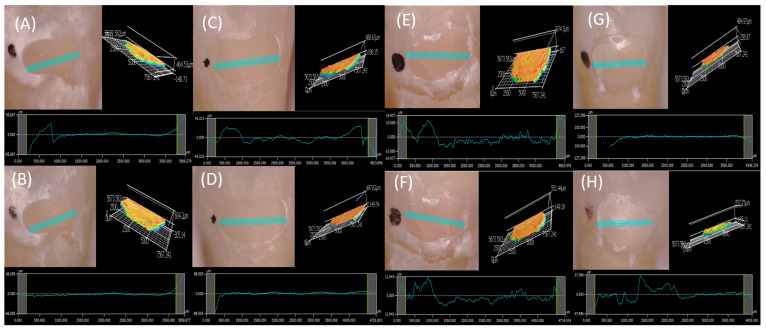
(**A**) Post restoration profile (1.202 mm) and surface roughness of Group A restoration. (**B**) Profile (1.202 mm) and surface roughness after 5 clinical years of Group A restoration. (**C**) Post-restoration profile (1.202 mm) and surface roughness of the Group D restoration. (**D**) Profile (1.202 mm) and surface roughness after 5 clinical years of the Group D restoration. (**E**) Post-restoration profile (1.202 mm) and surface roughness of the Group B restoration. (**F**) Profile (1.202 mm) and surface roughness after 5 clinical years of the Group B restoration. (**G**) Post-restoration profile (1.202 mm) and surface roughness of the Group C restoration. (**H**) Profile (1.202 mm) and surface roughness after 5 clinical years of the Group C restoration.

Figure 4 presents the surface profile and surface roughness measured using a 3D profilometer immediately post-restoration and after five clinical years. The results reveal an apparent deterioration in the surface profile of the Group C restoration shown in Figure 4G,H.

**Figure 5:**
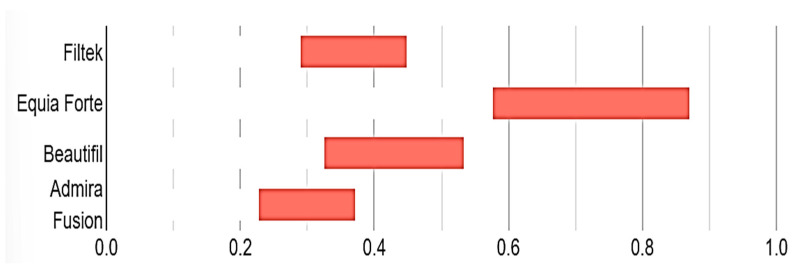
Confidence intervals for surface roughness measurements.

Figure 5 displays the confidence intervals for the averages of all examined materials. EQUIA Forte HT demonstrated a statistically significant difference compared to the other materials evaluated.

## 4. Discussion

The demand for esthetic restorations is rising due to the development of new and innovative restorative materials. The inherent properties of these materials, along with their ability to achieve a smooth surface, are fundamental for maintaining esthetic outcomes [32]. Surface roughness is a critical criterion for patient satisfaction and the aesthetic appearance of composite resins within the dynamic oral environment [33].

Eliminating surface irregularities in restorative materials is essential for their clinical longevity and aesthetic appeal. A smooth surface reduces plaque retention, thereby minimizing the risk of gingival irritation, surface staining, patient discomfort, and secondary caries [34]. However, the challenges posed by surface roughness in dental materials also create opportunities for advancement. Factors such as inadequate polymerization, poor oral hygiene, and thermal fluctuations in the oral environment can influence surface texture. Thermocycling, a widely used artificial aging technique, simulates the response of restorative materials in the oral environment and presents an area where further insights can drive significant improvements. Thermocycling, or thermal aging, is a method that exposes the material to a wide range of temperatures to determine the compatibility and strength of composite materials. Thermal aging has a notable impact on the roughness of composite-based materials, highlighting the importance of optimizing their properties for durability and esthetic stability [35]. Thermal cycling, through temperature variations, simulates the entrance of hot and cold substances in the oral cavity and shows the relationship of the linear coefficient of thermal expansion between the tooth and restorative material [36].

The 3D non-contact laser profilometer used in the present investigation offers a higher resolution than traditional mechanical stylus methods. The laser confocal technique was utilized as a viable alternative for non-contact and non-destructive surface measurements, thus addressing the limitations associated with the stylus method. For measurement precision, the laser profilometer offers a high accuracy for 3D topographical analysis, effectively measuring surface roughness and profile with reliable repeatability. However, it may be limited in its ability to detect extremely fine details. Scanning Electron Microscopy (SEM) is primarily qualitative and often requires complementary techniques such as energy-dispersive X-ray Spectroscopy (EDS) for quantitative surface analysis. In terms of applicability, SEM is limited in quantitative height measurements compared to profilometry. The laser profilometer eliminates the risk of surface damage caused by mechanical sensor contact, which could introduce errors in the results. The laser confocal sensor used in this study is equipped with both vertical and lateral scanning capabilities. This method overcomes the challenges posed by autofocus systems, which typically require the scanning unit to move with each sectioning step. Additionally, while traditional stylus measurements are conducted at a speed of 1 mm/s, the present 3D non-contact laser confocal system achieves measurement speeds of up to 3 mm/s, enabling in situ surface roughness assessment [37,38].

Patient comfort can be associated with a smooth and well-polished restoration in which a mean roughness of 0.50 µm can be detected by the patient [39]. In the present investigation, after five years of clinical use, all of the examined materials showed a linear increase in surface roughness, remaining within or below the 0.50 µm threshold, which is the level of roughness detectable by patients [39]. However, the hybrid glass (Equia Forte) exhibited a significant increase in surface roughness, which has significant implications for the longevity and patient satisfaction of these restorations. These findings align with previous studies, which have concluded that the surface topography of hybrid glass deteriorates over time, especially when exposed to varying environmental conditions, leading to a marked increase in surface roughness [40].

Previous studies have reported that water sorption and solubility in glass-hybrid restorative materials can reduce their mechanical properties, with surface coatings providing protection against initial water contamination [41,42]. Other studies have compared the mechanical properties and surface degradation of Equia Forte restorative materials with and without surface coatings, finding that minimal weight loss occurred in the surface-coated Equia Forte HT group [25,43]. In the present study, the Equia Forte HT material was handled according to the manufacturer’s instructions, with the restoration surface coated using the recommended coating material. The null hypothesis of this study was rejected due to the considerable difference in surface roughness observed with Equia Forte HT.

Previous studies have also established a significant positive correlation between average filler size and surface roughness [44,45]. Filler size and aging procedures have been shown to impact surface properties [45]. The surface roughness of composite resins is closely related to and influenced by the size of the filler particles [46]. In the present study, Admira ormocer restorations exhibited the smallest changes in surface roughness, followed by nanohybrid composites. This can be attributed to the type of filler; ceramic fillers, with their high abrasion resistance, confer superior polishability to the composite resin [47].

Further research is necessary to examine the clinical performance of such materials. While surface roughness remains a crucial aspect of successful materials, there are other factors that also contribute to the clinical effectiveness of such restorations. Materials must be exposed to different environmental changes that mimic the oral environment, such as brushing simulation and food/drink exposure. The importance of color stability, a key element of patient satisfaction, cannot be overstated and should be a focus of future research. Based on the results of the present study, the surface roughness of the hybrid glass ionomer Equia Forte HT exceeds the confidence intervals of the other tested materials. Despite this, glass ionomer materials may still be advantageous in specific clinical scenarios due to their ability to form adhesive micromechanical interlocking and chemical bonds with the tooth structure [48].

## 5. Conclusions

Within the limitations of the present study, the surface topography of all restorative materials changed after thermal aging. The ormocer material (Admira Fusion) exhibited the least changes in the average surface roughness and overall surface alteration. In contrast, the hybrid glass ionomer material (Equia Forte HT) showed a statistically significant increase in surface roughness, with noticeable surface alterations.

## Figures and Tables

**Table 1:** Type and composition of the examined materials.

Material (Group)	Type	Composition	Manufacturer	Lot
Admira Fusion x-base (Group A)	Ormocer	Ormocer, photoinitiators, pigments, barium aluminum borosilicate glass, pyrogenic silica (20–50 nm)	VOCO, Cuxhaven, Germany	2217143
Beautifil II (Group B)	Giomer restorative material	Bis-GMA, TEGDMA, multifunctional glass filler and S-PRG filler based on aluminofluoro-borosilicate glass	Shofu Inc., Kyoto, Japan	072277
Equia Forte HT. (Group C)	High-viscosity glass ionomer cement	Fluoro Alumino Silicate (FAS) glass, reactive silicate particle, high-molecular-weight polyacrylic acid.	GC, America Inc., Alsip, IL, USA	2106151
Filtek Supreme Ultra. (Group D)	Nanoparticle composite resin	Bis-GMA and TEGDMA, along with a high percentage of inorganic fillers, including nano-sized particles and radiopaque agents	3M, Saint Paul, MN, USA	NF27582

**Table 2:** Comparison between the examined materials using one-way ANOVA with post hoc Tukey HSD test.

Material	Post-Restoration		1st Year		3rd Clinical Year		5th Clinical Year	
	Mean	±SD	Mean	±SD	Mean	±SD	Mean	±SD
1. Group A	0.2465	0.1001	0.2835	0.1184	0.3128	0.1176	0.3356	0.1287
2. Group B	0.3910	0.2092	0.4116	0.2151	0.4152	0.1622	0.4529	0.1953
3. Group C	0.6280	0.2413	0.6938	0.2262	0.7217	0.3272	0.8484	0.3473
4. Group D	0.3226	0.0861	0.3574	0.1387	0.3630	0.1302	0.4038	0.1648
*p* value ^#^	<0.001 **		<0.001 **		<0.001 **		<0.001 **	
Material A vs. B	0.048 *		0.122		0.386		0.358	
Material A vs. C	<0.001 **		<0.001 **		<0.001 **		<0.001 **	
Material A vs. D	0.508		0.571		0.862		0.773	
Material B vs. C	<0.001 **		<0.001 **		<0.001 **		<0.001 **	
Material B vs. D	0.595		0.779		0.847		0.901	
Material C vs. D	<0.001 **		<0.001 **		<0.001 **		<0.001 **	

^#^ = * *p* < 0.05: significant; ** *p* < 0.001: highly significant.

## Data Availability

All data have been included in this study.
